# Being moved: linguistic representation and conceptual structure

**DOI:** 10.3389/fpsyg.2014.01242

**Published:** 2014-11-03

**Authors:** Milena Kuehnast, Valentin Wagner, Eugen Wassiliwizky, Thomas Jacobsen, Winfried Menninghaus

**Affiliations:** ^1^Centre for General Linguistics (ZAS)Berlin, Germany; ^2^Cluster of Excellence ‘Languages of Emotion’, Freie UniversitätBerlin, Germany; ^3^Department of Language and Literature, Max Planck Institute for Empirical AestheticsFrankfurt am Main, Germany; ^4^Helmut Schmidt University/University of the Federal Armed Forces, Experimental Psychology UnitHamburg, Germany

**Keywords:** being moved, emotion concepts, free word association, linguistic representation, prosocial feelings

## Abstract

This study explored the organization of the semantic field and the conceptual structure of moving experiences by investigating German-language expressions referring to the emotional state of being moved. We used present and past participles of eight psychological verbs as primes in a free word-association task, as these grammatical forms place their conceptual focus on the eliciting situation and on the felt emotional state, respectively. By applying a taxonomy of basic knowledge types and computing the Cognitive Salience Index, we identified joy and sadness as key emotional ingredients of being moved, and significant life events and art experiences as main elicitors of this emotional state. Metric multidimensional scaling analyses of the semantic field revealed that the core terms designate a cluster of emotional states characterized by low degrees of arousal and slightly positive valence, the latter due to a nearly balanced representation of positive and negative elements in the conceptual structure of being moved.

## Introduction

Feelings of “being moved” are emotional states experienced in situations as different as watching one's own child winning a prize at a school competition, listening to a favorite love song, or witnessing human misery after natural disasters. Whereas emotions like fear, anger, sadness, and happiness have received abundant experimental and theoretical attention, being moved is not yet well understood. For instance, although many languages provide lexicalized deverbal expressions for this feeling, such as *Rührung, or Ergriffenheit* (German), *ontroering* (Dutch), *commozione* (Italian), *kandoh* (Japanese), *terharu* (Indonesian), *rastrogannost*', *vzvolnovanost*' (Russian), *dirnutost* (Serbian), and *trognatost, umilenie* (Bulgarian), *being moved* is only rarely found on lists of emotion terms (Clore et al., 1987; Scherer et al., 2004). Researchers in the field of psychology have only recently turned their attention to the feeling of being moved (Tokaji, 2003; Konečni, 2005; Tan, 2008). Most of the interest in this topic is found among researchers studying emotional responses to artworks (see Tan and Frijda, 1999; Konečni et al., 2007; Oliver and Bartsch, 2010). However, many situations in everyday life are likewise readily described as moving. The present study therefore adopted a broader approach and did not specifically focus on art-reception. Its goal is to map the more general meaning of being moved. This includes references to experiences of art-reception, but does not cover the more specific discussions about being moved in rhetoric and aesthetics (cf. Konečni, 2005, 2011).

In English, an array of semantically similar terms such as feeling *moved*, *touched*, and *stirred* refer to this emotional state, and a corresponding set of adjectives such as *moving*, *touching*, and *elevating* can be used to describe and qualify specific triggers and situations in which this feeling occurs. German, a closely related language, has a similar set of terms such as *bewegt* (moved), *gerührt* (stirred), *berührt* (touched), and *ergriffen* (deeply moved, literally: seized) which are based on similar etymological and derivational patterns. (Throughout the text we use italics to refer to the German word forms and give English translations in brackets.)

In this exploratory study, we investigated German-language expressions which refer to states of being moved in order to elucidate the conceptual structure of this class of emotional states. Furthermore, we explored the relations between these different terms in order to identify core and peripheral members of the semantic field and their distinctive features. We applied a free word-association task, as this method allows us to tap into the conceptual structure of moving experiences and also to draw inferences about the organization of the semantic field (Deese, 1962, 1965; Nelson et al., 2000; Jacobsen et al., 2004). Word associations reflect the lexical knowledge people acquire through world experience by means of words and the relations between them as units of the linguistic system. Information obtained by word associations thus always comprises linguistic and conceptual knowledge (Nelson et al., 2004; Santos et al., 2011).

Lexical items reflect the underlying representation of a concept not directly, but rather mediated through their linguistic representation, which encompasses language-specific information such as phonological, morphological, and syntactic properties. Jackendoff (2002, 2007) argued that the abstract semantic structure of lexical entries yields a good approximation of the conceptual structure of the entities to which they refer. The abstract semantic structure motivates the syntactic behavior of words by means of interface rules between conceptual and linguistic representations. Targeting the emotional state of being moved, we need to take into account that the lexical expressions which refer to this state in German, English, French, and many other languages are primarily verbs, e.g., *to move, to touch, to stir*, and their participles or verbal nouns.

Verbs which refer to what people experience in moving situations belong to the semantic class of psychological verbs. The thematic structure of such verbs involves a relation between an Experiencer and a Stimulus which is conceived of as the cause of the emotional or mental state (Dowty, 1991). Research on the relation between underlying thematic roles and causation types has shown that in the case of psychological predicates, the occurrence of a particular mental or emotional state is causally related to a stimulus but finally depends on the appraisal of the situation by the feeling and thinking subject (Arnold, 1960; Croft, 1993; Primus, 2006; Scherer, 2009).

Regardless of this important qualification, verbs designating the emotional experience of being moved in German and other European languages are transitive verbs with Stimulus and Experiencer as underlying thematic roles. In unmarked active sentences (such as *The speech moved him deeply*), the subject position is filled with the Stimulus of the emotional state, while the Experiencer occupies the direct object position. This realization of the arguments is linked to the event structure of these verbs which designate change-of-state events. The dynamic event type sets these verbs apart from other emotion verbs such as *love* or *envy* which designate emotional states which remain stable for a certain time span (Rothstein, 2004). Such verbs, also classified as Subject–Experiencer verbs (Levin, 1993), express non-causative meaning. They represent emotional states as attributable to the sentient individual and directed toward an object. The basic semantics of verbs like *love*, *envy*, and *fear* thus contrasts with the strong causative meaning of Subject-Stimulus verbs like *move*, *touch*, and *shatter*, with the latter highlighting the role of the external stimulus for the experienced non-volitional and instantaneous change of state (Levin and Rappaport Hovav, 2005).

Present and past participles inherit the thematic structure of the underlying verbs but focus on different aspects of the mental representation of the designated emotional episode. Syntactically, present participles function as adjectives which designate properties of the subject (Borer, 1990; Brekke, 1988). In terms of temporal structure, present participles refer to a state of affairs and to those features of the situation which are sufficient to induce the change of state which is constitutive of the lexical meaning of dynamic psychological verbs. Past participles, by contrast, focus on the resulting state of affairs, designating the experienced emotional or mental state. Our study takes advantage of these grammatical properties by using them as a window into the conceptual structure of the emotional state of being moved.

## Materials and methods

Following a tradition of emotion research (e.g., Fehr and Russell, 1984; Russell and Fehr, 1994; Fontaine et al., 2002; Storm and Storm, 2005) which assumes that linguistic expressions like *a moving speech* and *I was deeply touched* activate the representation of the respective emotion concepts, our word-association study explored the following questions: (1) What are the prototypical antecedents, feeling qualities, and types of expressive behavior in moving situations which can be revealed through an analysis of the associative conceptual structure of words used to designate such eliciting situations and corresponding emotional states? (2) What is the organization of the semantic field of being moved, as represented through the relations between these emotion terms, and which affective properties might reliably differentiate between core and peripheral instantiations of the state of being moved?

### Participants

We recruited 815 participants in the waiting rooms of several citizen centers in Berlin. They volunteered for the pen-and-paper study and gave their informed consent. Six hundred and ten participants (307 female) were native German speakers. We concentrate on the data from the native German speakers, for two reasons. First, there is linguistic and ethnological evidence that emotion terms and their uses are culturally entrenched (Wierzbicka, 1999; Parkinson et al., 2005). Second, we had no information about levels of linguistic proficiency or the non-native German speakers' potential to fully conform to an experimental design based on the grammatical features of German participles.

The mean age of the sample was 37 years, with ages ranging from 18 to 80; four participants did not report their age. There was no age difference between female and male participants, *t*_(604)_ = −0.924, *p* = 0.36. With respect to levels of education, the sample exhibits a balanced structure, with 46.4% of the participants having a university-level education or a general qualification for university entrance. Female (47.1%) and male (45.7%) participants had similarly high levels of education. Four participants did not report their educational degree.

### Materials

The stimuli comprised several German verbs frequently used in an affective-evaluative manner to refer to moving experiences. The primary targets *bewegen* (to move), *berühren* (to touch), *rühren* (to stir) and *ergreifen* (to move deeply; literally: to seize) were selected according to their core lexical meaning and synonymy relations (for a similar selection procedure, see Shaver et al., 1987). Additionally, we collected data for the present and past participles of the verbs *aufregen* (to excite), *erschüttern* (to shatter), *erheben* (to elevate), and *packen* (to grip). The choice of these control words was motivated by a twofold reason. First, the control terms selected share with “being moved” the following three features: (a) they place a strong conceptual emphasis on the subjective feeling component of emotions; (b) their use covers a broad range of eliciting events/scenarios; (c) they all appear to involve multiple emotional ingredients and hence are of a complex nature. Second, we hypothesized that these terms would differ from the core being moved/being touched terms with regard to the important dimensions of valence and arousal. With respect to affective valence, we predicted *shattering* to be more unambiguously negative, and *elevating* to be more unambiguously positive as compared to the being moved terms. Regarding arousal, we assumed *exciting* and *gripping* to be of higher arousal than the core *being moved* terms. Since the German participles *berührt* (touched) and *gerührt* (stirred) depict states of fairly low arousal, we did not find an emotion term likely to be of lower arousal, while also conforming to the three criteria mentioned above. The inclusion of “gripping” was additionally motivated by the specific lexicalization pattern of the German term *packend.* The source domain of this metaphorical emotion term is directly synonymous with that of *ergreifend* (*deeply moving*), both verbs, *packen* and *ergreifen*, depicting the events of gripping, seizing or grasping. This combination of commonalities and differences made the four additional terms attractive for a multi-dimensional scaling and mapping of the core being moved terms.

We used the present and past participles derived from the eight selected verbs (e.g., *bewegend* [moving] and *bewegt* [moved], respectively) as primes in the word-association task. Thus, we had 16 primes classified by the two crossed factors Grammatical Form (present vs. past participle) and Emotion Verb (*bewegen, rühren, berühren, ergreifen, erschüttern, aufregen, packen, erheben*). A list of the test items in German and their approximate English translations is given in Table 1. We are aware of the difficulties of translating these terms and of their high variability when used in different contexts and collocations (Hurtado de Mendoza et al., 2010; Kayyal and Russell, 2013). The English terms used are approximate translations of the German verbs; they rely in a very similar fashion on metaphorical uses of the underlying physical movement types.

**Table 1:** **Experimental stimuli: descriptive statistics**.

**Primes**		**Participants**	**Number of individual entries**	**Number of different words**	**Subjective frequency**		**Valence**	**Arousal**
**English translation**	**German**	***N***	***N***	***N***	***M***	***SD***	***M***	***M***
**PRESENT PARTICIPLES**								
Moving	(*bewegend*)	35	93	18	4.5	1.4	0.84	3.15
Touching	(*berührend*)	48	159	24	4.1	1.7	0.99	3.22
Stirring	(*rührend*)	40	103	22	3.6	1.6	0.49	3.08
Deeply moving	(*ergreifend*)	39	87	15	3.5	1.7	0.84	3.20
Elevating	(*erhebend*)	37	67	12	2.7	1.5	2.32	3.05
Shattering	(*erschütternd*)	36	67	15	3.3	1.5	−2.00	3.89
Gripping	(*packend*)	32	49	11	4.1	1.4	1.04	3.39
Exciting	(*aufregend*)	36	76	18	4.6	1.3	0.24	3.77
**PAST PARTICIPLES**								
Moved	(*bewegt*)	34	105	22	4.5	1.4	0.75	3.45
Touched	(*berührt*)	41	100	17	4.2	1.7	0.90	3.23
Stirred	(*gerührt*)	40	103	19	4.1	1.5	0.61	3.22
Deeply moved	(*ergriffen*)	36	81	13	3.4	1.8	0.26	3.45
Elevated	(*erhoben*)	38	36	9	2.1	1.3	1.58	3.29
Shattered	(*erschüttert*)	42	88	20	2.4	1.4	−1.17	3.41
Gripped	(*gepackt*)	39	71	16	3.0	1.7	1.12	3.49
Excited	(*aufgeregt*)	37	77	19	4.4	1.3	−0.01	3.71

Subjective frequency mean estimates (1 = almost never to 7 = very often); valence and arousal means based on the BAWL-R scores (Võ et al., 2009) of elicited associations.

All the selected verbs except *aufregen* (to excite) are conceptually polysemous in that they have both a physical interpretation and an emotional interpretation. The frequency counts available from German online corpora such as DWDS (Berlin-Brandenburg Academy of Sciences, www.dwds.de) are computed for the main lexicon entry by accumulating all morphological instantiations of a verb, regardless of the relevant semantic distinctions in polysemous verbs. In order to obtain a measure of the subjective frequency of the selected verb forms as emotional terms, we asked the participants to fill in a 7-point Likert scale (1 = *almost never* to 7 = *very often*) to indicate the frequency with which they use the stimulus participles as emotion words in their daily communication.

### Procedure

Participants were asked to concentrate on the stimulus item and to write down all words which came to their minds within a time span of 60 s. They were explicitly instructed not to build associative chains, but to focus on the stimulus item. Each participant received only one stimulus term, e.g., *emotional berührt* (emotionally touched), to avoid a confounding of associations through activation of semantically related stimuli. Furthermore, each of the 16 participles was presented together with the adverb *emotionally* in order to prime the psychological rather than the physical interpretation. The presentation of the participles was counter-balanced.

Subsequently, we collected demographic data including gender, age, native language, foreign language proficiency, and professional occupation. Additionally, we used a 4-point scale (1 = *never* to 4 = *very often*) to ask the participants about the frequency of their engagement in cultural activities such as going to the cinema or theater, playing an instrument, reading books or magazines, and attending exhibitions and concerts. Finally, participants were asked to estimate the frequency with which they use the emotional term under scrutiny and five other words which we used as reference points.

## Results

The participants generated a total of 3891 entries, which included words and phrases like *Freude* (joy), *traurig* (sad), *Beziehung* (relationship), *weinen* (cry), *Ende einer Beziehung* (end of a relationship), and *gute Laune* (to be in a good mood). This figure also includes illegible entries and words written twice by the same participant.

For the subsequent analyses, the data were preprocessed as follows. The entries obtained were corrected for spelling and grammatical number. Plural forms like *Beziehungen* (relationships) were recoded in their singular form (*Beziehung*, relationship). However, in cases where plural forms are more commonly used to express generic meaning, the plural forms were preferred. Thus, the entry *Kind* (child) was recoded as *Kinder* (children).

Only words listed by at least three participants in response to the individual primes were included in the further analyses (for a similar cut-off procedure, see Van Goozen and Frijda, 1993; Russell and Fehr, 1994; Istók et al., 2009). This procedure reduces variability due to idiosyncratic uses. After the pre-processing, 93 different words (associations)—altogether representing 1362 individual entries—constituted the final data set.

Table 1 shows the number of entries, and the number of different words per prime, as well as the mean subjective frequency estimates. Present and past participles did not elicit significantly different numbers of entries [701 vs. 661; χ^2(1)^ = 1.18, *p* = 0.28]= or words [57 vs. 66; χ^2(1)^ = 0.66, *p* = 0.42]. Participants wrote down between 1 and 19 entries (*M* = 6.49, *SD* = 2.95). They were most fluent in their responses to the present participle *berührend* (touching; 159 entries, 24 words), and to the past participle *bewegt* (moved; 105 entries, 22 words). Overall, the number of entries was positively correlated with the subjective usage frequency of the stimulus items, *r*_(586)_ = 0.18, *p* < 0.001.

### Conceptual structure of being moved

Since we were primarily interested in the conceptual structure of the emotional state of being moved, we analyzed the associated words with respect to linguistically and conceptually anchored knowledge types which might allow us to make inferences about prototypical triggering situations and feeling qualities. We applied the taxonomy of basic knowledge types proposed by Wu and Barsalou (2009). This taxonomy distinguishes between two main types of associations: those reflecting features of the primes as entities of the linguistic system such as synonyms and antonyms or phrases and idioms, and those reflecting conceptual knowledge in terms of taxonomic relations, situation features and introspective features (see also Santos et al., 2011 for experimental evidence and discussion). Situation features comprise knowledge about prototypical participants, location, time, manner, and actions. Introspective features comprise semantic knowledge about affect, positive or negative evaluation, and causal relations. For the present purposes, we collapsed linguistic knowledge types and taxonomy relations into one category. There is evidence that ontological taxonomies of superordinate concepts and their subcategories closely mirror semantic structures in the mental lexicon conceived of as relations between hyper- and hyponyms (*emotion: fear, joy, sadness*), or between cohyponyms expressing synonymous meaning like *sad, gloomy*, and *distressed* (Lyons, 1977, p. 271; Murphy, 2002, 2003).

Two raters classified the associated words (*N* = 93) as representing either linguistic knowledge or situational and introspective features of the conceptual representation. The inter-rater reliability was found to be κ = 0.92, *p* < 0.001, showing a very good agreement. The remaining differences were resolved by discussion.

Both participle types elicited word associations reflecting taxonomic relations such as the superordinate *Gefühl* (feeling/emotion) and synonyms like *bewegend* (moving) or *gerührt* (touched; *N* = 9). In German, the present participles under investigation are regularly used attributively in constructions such as *ein erhebendes Gefühl* (literally: an elevating feeling). Expressions of this type refer to the emotional state of elevation as a hyponym of the superordinate concept *Gefühl* (feeling/emotion).

Words such as *reunion, books*, and *friends* were rated as designating situation features, as they refer to prototypical occasions and participants in moving situations (*N* = 45). Words such as *joy, sad*, and *laughing* were rated as referring to the experienced emotional state or properties of the experiencer, thus representing introspective features (*N* = 39). Twenty-nine words were elicited by both participle types. They were equally distributed into the categories of situational (*N* = 14, e.g., *death, film, children*) and introspective (*N* = 15, e.g., *sadness, love, tears*) features.

Based on the assumption that present and past participles focus on the emotion antecedent and on the resulting emotional state, respectively, we analyzed the distribution of the associated words taken to represent conceptually relevant knowledge types. A chi-square test revealed a significant difference between participle types, χ^2^_(1)_ = 5.70, *p* = 0.023. Present participles triggered more words designating situation features than introspective ones (65 vs. 35%), while past participles showed the inverse distribution of knowledge types (42 vs. 58%).

**Figure 1:**
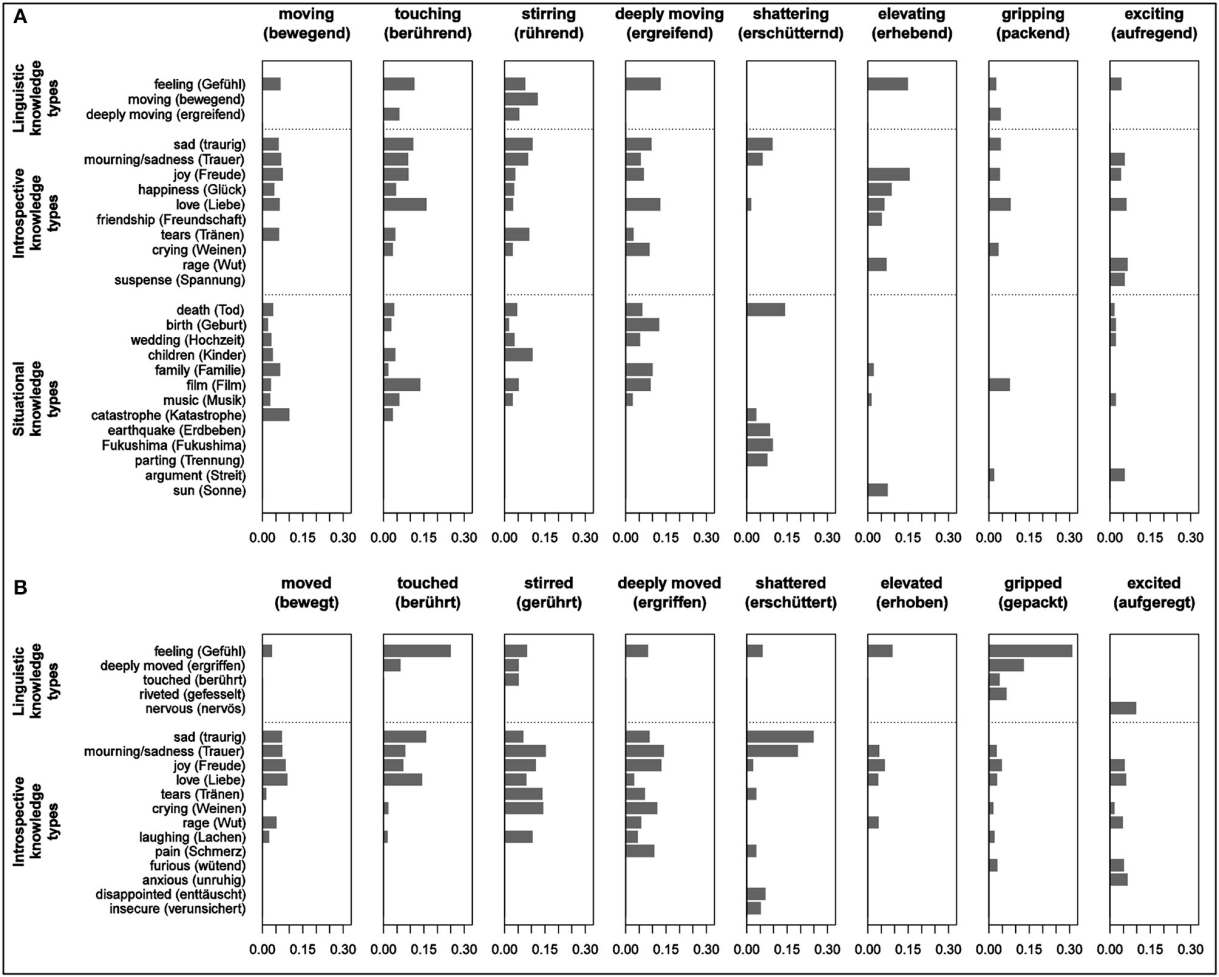
**Cognitive Salience Index of the most salient associations elicited by present participles (A) and by past participles (B)**. Only associations which reached a CSI ≥ 0.05 in at least in one subsample are plotted. The associations are organized according to the three knowledge types: taxonomic relations, situational features, and introspective features.

In order to arrive at a more precise analysis of the associative structure of the investigated verb forms, we computed the Cognitive Salience Index (CSI) of the word associations elicited by each individual prime (Sutrop, 2001). The rationale behind the CSI = *F*/*N* ^*^ *mR* is that relevant terms are accessible for most of the participants (relative frequency, F/N) and those terms tend to be retrieved more quickly and therefore tend to be listed among the first entries (mean rank, *mR*). The CSI yields a value between 0 and 1, with greater values corresponding to greater cognitive salience. Figure 1 shows the word associations which reached at least a CSI value of 0.05 in one of the individual samples, ordered by knowledge type.

With respect to linguistic knowledge (upper portion of Figures 1A,B), all verbs under scrutiny elicited the term *Gefühl* (feeling/emotion). The availability of stable collocations—be they adjective phrases with present participles of the type *ein erhebendes Gefühl* (an elevating feeling), or resultative constructions of the type *ein Gefühl hat mich gepackt* (I was gripped by a feeling)—seems to have increased the CSI values of the word *Gefühl* as compared to the cases where it was elicited only as the superordinate term. With regard to synonymy relations, we found that participants perceived the verb *ergreifen* (to move deeply) to be closely related to *rühren* (to stir) and *berühren* (to touch). *Bewegend* (moving) obtained a high CSI value as a synonym of *rührend* (stirring).

With respect to words that reflect introspective features, we found considerable parallels between present and past participles. In both conditions, the emotion terms *Liebe* (love), *Freude* (joy), *Trauer* (mourning/sadness), and *traurig* (sad) consistently had high CSI values. There was a consistent prominence of both joy and sadness in the affective spectrum of all target verbs which designate moving experiences in the narrower lexical sense, that is, *bewegen* (to move), *berühren* (to touch), *rühren* (to stir), and *ergreifen* (to move deeply). In contrast, the word associations obtained for the remaining four verbs did not include the two antithetical emotions of joy and sadness. The associative range for *erschüttern* (to shatter) was dominated by negative affective terms like *traurig* (sad), *Trauer* (mourning/sadness), and *enttäuscht* (disappointed), while the participle forms of *erheben* (to elevate) were clearly associated with positive affective terms like *Freude* (joy) and *Glück* (happiness). The low CSI values for *Trauer* (mourning/sadness) and *Traurigkeit* (sadness) indicated the lower relevance of these affective states for how the participants conceived of exciting or gripping events. Terms designating facial and bodily expressions of affective states like *Weinen* (crying), *Tränen* (tears), *Lachen* (laughing), or *Lächeln* (smiling) also reached a high level of salience within the knowledge domain of introspective conceptual features. In addition to *Lachen* (laughing), *Weinen* (crying), and *Tränen* (tears) appeared to be among the most important introspective features for the verbs *rühren* (to stir) and *ergreifen* (to move deeply). Their relevance in the conceptual makeup of *rühren* (to stir) is also reflected in the idiomatic expression *zu Tränen gerührt sein* (to be moved to tears). Fuelled by conceptual and linguistic associative strength, the term *Tränen* (tears) reached its highest CSI value for the past participle *gerührt* (stirred).

In contrast to the significant overlap regarding introspective conceptual properties, past and present participles clearly differed with respect to the associated words representing situational properties. While the present participles elicited a broad range of such words exceeding a CSI of.05, past participles only yielded situation concept-related word associations that remained below this level. *Geburt* (birth) and *Tod* (death) reached only CSI = 0.041 for the subsamples *gerührt* (stirred) and *bewegt* (moved), respectively (see Table S1, Supplemental Material).

Events of personal relevance such as birth, death, and marriage, along with their prototypical actors such as family, children and friends, appeared as basic examples of moving and touching situations. Words referring to emotion eliciting artworks such as *Film* (film) and *Musik* (music) turned out to be further key elements in the conceptual structure of the core being moved terms. Words referring to these two prototypical trigger types obtained high CSI values and were consistently represented in the associative range of the four primary verbs.

Death and related situation features such as loss, funeral, and mourning belong to the central event types in the conceptual structure of moving situations. At the same time, the terms *Geburt* (birth) and *Hochzeit* (wedding) that designate profoundly positive events have comparable relevance for the generic representation of moving and touching situations. This distribution of positive and negative elicitors points toward finer differences in the conceptual structure of the core terms under scrutiny. While the situation features of deeply moving (*ergreifend*) events were dominated by positive elicitors, those of moving (*bewegend*) events also included some negative elicitors, like large-scale natural or human-made disasters.

### Dimensional modeling of the semantic field

In order to explore the organization of the semantic field of the being-moved terms (along with the control terms) investigated in this study, we estimated the similarities between the 16 primes based on the frequency of the word associations obtained. As a similarity measure, we calculated the overlapping coefficient (OC) of the primes according to Marx (1976a,b; see also Inman and Bradley, 1989). The OC is computed by summing up all the lower relative frequencies in the two distributions for each word *j*: *OC*(*A, B*) = ∑min[*p*(*A*_*j*_), *p*(*B*_*j*_)] (we provide the complete similarity matrix as Table S2 in the Supplementary Material). To examine the internal consistency of the data, we compared the similarity matrices of the present and past participles by performing the Mantel test (Mantel, 1967; Schneider and Borlund, 2007). We found a significant correlation (*r* = 0.667, *p* = 0.02; ρ = 0.618, *p* = 0.04) between the similarity matrices of the subsamples comprising present and past participles.

The complete similarity matrix was entered into the multidimensional scaling procedure PROXSCAL (Commandeur and Heiser, 1993). To achieve an optimal approximation of the fine data structure, we used Torgerson start as recommended by Borg and Groenen (2005, p. 556) and a spline transformation to smooth curve estimations (Ramsay, 1988; Groenen et al., 2000). As a goodness-of-fit indicator for MDS solutions with different numbers of dimensions we used the index D.A.F. (Dispersion Accounted For) derived from the normalized raw stress (Borg and Groenen, 2005, pp. 247ff). Compared to a one-dimensional solution (D.A.F. = 0.92), the two-dimensional solution yielded a clearly higher value (D.A.F. = 0.97), whereas adding a third dimension increased the goodness-of-fit value only marginally (D.A.F. = 0.98). Based on a scree-test, a two-dimensional solution was selected.

**Figure 2:**
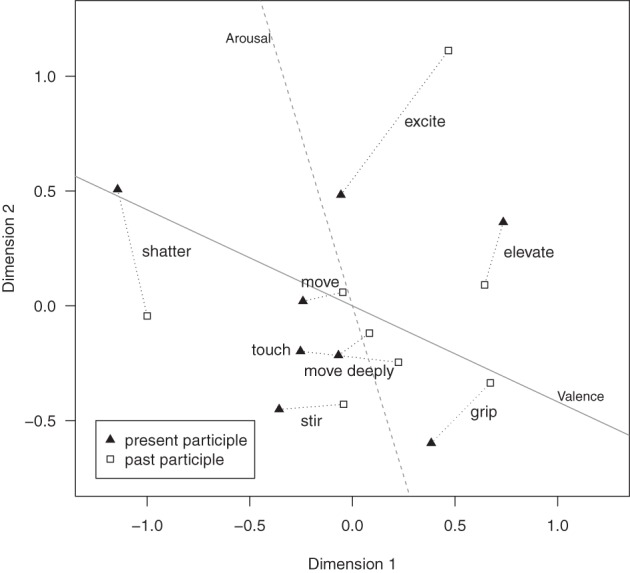
**Metric multidimensional scaling of the 16 participles**. Each participle pair (e.g., moving–moved) is connected by a dotted line. The solid line and the dashed line represent the fitted BAWL-R based valence and arousal dimensions, respectively.

The MDS solution (see Figure 2) may be reasonably interpreted as reflecting valence (Dimension 1) and arousal (Dimension 2). The participles of *erheben* (to elevate) and of *erschüttern* (to shatter) mark the positive and negative extremes in the valence continuum, while the participles of *aufregen* (to excite) and *rühren* (to stir) feature as the opposite extreme values of the arousal continuum. In order to validate this interpretation, we decided to fit independently determined valence and arousal values of the primes into the resulting configuration (Borg and Groenen, 2005, p. 77). The *Berlin Affective Word List Reloaded* (BAWL-R; Võ et al., 2009) provided valence and arousal ratings for about 80% of the words obtained through our study. For each of the 16 primes, we calculated valence and arousal means weighted by frequency of the words to adjust cell totals to the unequal sample sizes (see Table 1). We regressed these valence and arousal values on the coordinates obtained in the MDS.

The correlation (*r* = 0.87) obtained between the predicted and the BAWL-R-derived valence values indicates that the vector fitted well in the configuration for the 16 participles. The fitting of the arousal vector was still good, although clearly lower (*r* = 0.61). Additionally, these two vectors were aligned nearly orthogonally to each other, demonstrating the independence of the two dimensions.

Apart from the interpretation of the dimensions in the MDS solutions, we analyzed the configurations obtained with respect to the distance relations between the primes. Although based on data obtained from two subsamples, the present and past participles of the verbs *bewegen* (to move), *berühren* (to touch), *rühren* (to stir), and *ergreifen* (to move deeply) clustered together in the represented semantic space. Moreover, all eight being-moved terms were grouped closely to the zero point of the valence axis, reaching a slightly positive position overall.

The participle forms of the remaining four verbs were located in the periphery of the semantic space (*aufregen* [to excite], *erschüttern* [to shatter], *erheben* [to elevate]) or at least at a greater distance from the core items (*packen* [to grip]). In terms of arousal, the peripheral terms were clearly different from the core terms. The core verbs *bewegen* (to move), *berühren* (to touch), *ergreifen* (to move deeply), and *rühren* (to stir) represented a group of emotional states with descending degrees of arousal, ranging from moderate (*bewegt* [moved]) to low (*gerührt* [stirred]) arousal. The present and past participles of the core verbs featured very similar levels of arousal. In contrast, the participles of the peripheral terms displayed substantial differences in arousal. The present participles of the verbs *erheben* (to elevate) and *erschüttern* (to shatter), which marked the positive and the negative valence poles in the MDS configuration, exhibited higher levels of arousal than the corresponding past participles. The opposite distribution was obtained for the forms of the verbs *packen* (to grip) and *aufregen* (to excite), the latter designating prototypically high-arousal emotional states.

## Discussion

Our exploratory study of the emotion terms designating moving experiences utilized the linguistic properties of eight psychological change-of-state verbs and the grammatical properties of their present and past participles as point-of-view markers: present participles focus on the change-eliciting stimuli, whereas past participles focus on the experiencer.

The free associations elicited by these primes yielded a nuanced picture of the situational and introspective knowledge types which underlie the conceptual structure of the core German being-moved verbs—*bewegen* (to move), *berühren* (to touch), *rühren* (to stir), and *ergreifen* (to move deeply). In addition to highlighting important emotion antecedents, the associative range of the present participles replicated the specific pattern of oppositely valenced feeling elements prominent in the associative structure of the past participles. As reported above, the results for both types of participle showed a substantial overlap. Given the between-subject design of our study, this can be considered to be partial replication based on two separate datasets. The internal consistency of the data allowed us to model the semantic field of moving experiences along valence and arousal as a set of psychologically pertinent affective dimensions (Yik et al., 1999; Stanley and Meyer, 2009).

With respect to prototypical situations which trigger moving emotional episodes, we found three main elicitor types. The first elicitor type comprised significant life events such as births, deaths and weddings and their prototypical protagonists—family members and friends. The second elicitor type comprised art stimuli, most prominently represented by the high cognitive salience of the word associations *film* and *music*. Both of these elicitor types include events of different valence, instantiated either overtly, by death and birth, or covertly, as film and music can be either sadly or joyfully moving. At first glance, the third elicitor type—earthquakes and other calamities—appears to be limited to emotionally negative responses. However, natural catastrophes are not moving in themselves, but only through our understanding of their tremendous impact on the lives of the people affected. Witnessing human tragedy, either directly or through media coverage, stirs up empathy and solidarity with the victims, which may ultimately motivate prosocial behavior. Therefore, these elicitors also support a spectrum of negative and positive feeling components.

Comparing the occasions and protagonists which our German participants conceived of as moving, we found significant overlap with elicitors discussed by Konečni (2005, 2011) and Tan (2009), namely, artworks such as film and pieces of music, but most importantly significant life events such as births, deaths, and weddings. These events with high personal and social significance connect the experiencers to emotionally salient types of social bonds of different scales such as family, friendships, or nations. Many moving artworks, for example, dramas, films, novels, and poems, make use of similar social scenarios and the concomitant emotion scripts. What the artful representation might add to how these scenarios are experienced as moving—and by virtue of which means non-representational art forms, particularly music, can likewise strike us as emotionally moving—lies beyond the scope of the present study (but see Kivy, 1999, for a discussion of moving instrumental music). Applying a language-based association task, we did not obtain data with specific reference to acts of generosity and sacrifice discussed in other studies (Konečni, 2005, 2011), although we intuitively perceive such events as very moving.

In order to determine the affective nature of being moved, we investigated the semantic field of the 16 primes, conducting a metric MDS. The obtained two-dimensional solution was evaluated with respect to dimensional models of affective meaning (Osgood et al., 1975) and dimensional emotion theories (Yik et al., 1999; Fontaine et al., 2007). We interpreted the two dimensions as corresponding to the psychologically pertinent factors of valence and arousal, based on the valence dichotomy between the predominantly positive verb *erheben* (to elevate) and the negative verb *erschüttern* (to shatter), as well as based on the arousal-related dichotomy between the high-arousal verb *aufregen* (to excite) and the low-arousal verb *rühren* (to stir). The finding that the core terms *bewegen* (to move), *berühren* (to touch), *rühren* (to stir), and *ergreifen* (to move deeply) appeared to be of neutral valence, clustering around the zero point, needs to be understood in the light of the conceptual analysis of moving experiences presented above. The analyses are based on data across participants. Participants might show some variation in the mental representations of the being moved terms due to their individual experiences. As a result, no inference on the valence of individual episodes of being moved can be made based on our data. Specifically, the equal prominence of positive and negative word associations obtained in response to the being-moved terms is likely to have leveled the mean valence score. Figure S1 (Supplementary Material) illustrates the bimodal distribution of the words. The moderate-to-low arousal values are, moreover, corroborated by observations in the literature according to which the emotional state of being moved entails low action tendencies (Tan, 2009). Despite the fact, that the actual MDS solution depends on the specific terms and their relation as fed into the procedure, the dimensions are readily interpretable as representing valence and arousal. This conformity to the most fundamental distinction of emotion theory suggests that the solution is not arbitrary.

Notwithstanding the importance of the dimensions of valence and arousal for mapping the emotion terms under scrutiny, we do not imply that these dimensions exhaustively account for the emotional states designated by these terms. Rather, we conceive of being moved as a discrete emotional state that has a unique quality, and featuring cognitive, expressive, physiological, motivational, and subjective feeling components (Kleinginna and Kleinginna, 1981; Scherer, 2005; Grandjean and Scherer, 2008). While the terms investigated in this paper all place a particularly strong focus on how a person subjectively feels about a given emotion eliciting event (being moved, touched, shattered, etc.), some of the word associations obtained also have a clear bearing on expressive and physiological emotion components (e.g., laughing, tears). Moreover, sadly and joyfully moving episodes are likely to involve the cognitive appraisals characteristic of the emotions sadness and joy. Drawing on these two key emotional ingredients—and on the prototypical eliciting scenarios such as significant relationships and critical life events—we suggest that episodes of being moved are experienced as particularly significant, or meaningful, emotional episodes of a joyful and/or sad type. Moreover, from the almost equal preeminence of sadness and joy we found for episodes of being moved, several crucial questions arise: (1) What constraints have to be met in order for episodes of sadness and joy to be sadly or joyfully moving rather than just sad or joyful? (2) Presupposing that select episodes of joy and sadness converge on the character of being emotionally moving, is the state of being moved then only a subcategory of joy and sadness rather than an emotional state in its own right? Furthermore, do feelings of sadness or happiness form a blend with feelings of being moved in respective episodes, or do both merely co-occur in such episodes in another fashion yet to be determined? While the granularity of our data does not place us in a position to provide straightforward answers to these questions, we still feel encouraged to discuss them in the light of our results, as follows:

(1) Since our free-association data revealed a specific set of distinctive eliciting scenarios, there follows a clear rule of constraint: episodes of both joy and sadness appear to be eligible for being moving only in cases where they are responses to critical life and significant relationship events (births, weddings, funerals, separations, reunions, and witnessing one's children going through important milestones of their lives), to the plight of people following catastrophes, or to artworks that in one way or another bear on one of these scenarios. Moreover, extending a previous finding that being moved promotes generosity in charitable donations (Stel et al., 2008), we suggest that most of the eliciting scenarios identified by our study indicate a *strong significance of prosocial feelings* of bonding, attachment, and empathy for states of being moved. For example, birth is a moving event because it blends the pathos of new life with notions of affiliative bonding and parental responsibility, and funerals are moving only to the extent that they honor the deceased and revitalize both past social bonds and current ones. We do not imply that a strong bearing on prosocial feelings provides a *sufficient* condition for moving instances of several prototypical emotions, but it may well constitute a *necessary* constraint. Many happy and sad events fail to conform to the substantial constraints for moving episodes identified in the preceding paragraph; that is, they are neither critical life events nor natural catastrophes, nor are they tied to prosocial bonding. We suggest that episodes of being moved are experienced as particularly significant, or meaningful, emotional episodes of a joyful and/or sad type only because and to the extent that they meet these requirements of scenario specificity and of the (however discreet) involvement of prosocial norms and feelings.

Considered in this way, the character of “movingness” is not by definition included in the concepts of joy and/or sadness, but rather represents an additional feature found in highly select episodes of sadness and joy. Moreover, a variety of moving experiences occur outside the confines of happy or sad episodes. For instance, witnessing acts of great generosity and/or courage can be emotionally moving without concomitant feelings of joy or sadness.

(2) Concerning the question of whether feelings of being emotionally moved are only special modifications of sadness, joy, and other emotions (given the premise that they occur in conjunction with these emotions), or whether being moved constitutes a discrete emotion in its own right, we adopt a cautious stance. To the extent that *all* concepts can be considered selective mental constructs highlighting specific points of view or foci of perception (Barsalou, 2012), we tend to consider it equally legitimate (a) to focus on the feeling of being moved as experienced in a variety of different contexts and accordingly view it as an emotional phenomenon in its own right, or (b) to emphasize that barely any episode of being moved is only an episode of being moved and unrelated to concomitant emotions, and accordingly consider being moved as only a modifying feeling that adds a special flavor to a variety of very different emotions. Notably, the special flavor of being moved apparently feels the same—or at least sufficiently similar to be referred to by the same term—across different emotions, and it therefore constitutes an emotional phenomenon on its own. In any event, on the basis of the present data, we cannot safely decide between the alternative accounts of being moved sketched above.

Of course, many other interesting research questions pertaining to the state of being moved lie outside the scope of the present study. We would be very much interested in the mental chronometry and the functional role of being moved, as well as in the neural structures subserving it. These issues must be left to future research.

The present study has investigated the notion of being moved through the prism of the German language system. We are aware of the limitations that the scope of our study imposes on generalizations to other languages and cultures (Wierzbicka, 1999; Hurtado de Mendoza et al., 2010). Conceptualizations of the emotional state of being moved are highly likely to show some variation across languages and cultures. At the same time, we do notice conceptual similarities even across different families of languages. Thus, Germanic and Slavic languages use different word forms to express a focus on elicitors, or on the state of being moved. For example, several Slavic languages feature pairs of abstract nouns which distinguish the quality/capacity to move someone (*dirljivost* in Serbian, *trogatel'nost'* in Russian, and *trogatelnost* in Bulgarian) from the resulting state of being moved (*dirnutost, rastrogannost'*, *trognatost*). This distinction can be readily projected onto what the German and English languages distinguish through the grammatical forms “moving” (present participle) vs. “moved” (past participle).

In closely related languages such as German and English, there are good indications for strong conceptual similarities. The English terms *moved* and *touched* appear to broadly and closely correspond to four German expressions: *bewegt, ergriffen, gerührt*, and *berührt*. Specifically, the two languages converge in an idiomatic expression referring to shedding tears as a characteristic physiological expression of being moved: *to be moved to tears* and *zu Tränen gerührt sein*. In addition, Ferstl et al.'s (2011) study has rated the English psychological verb *to move* as having on average only slightly positive valence (0.3 on a scale of −3 to 3).

We assume that the subjective feeling components and the emotion antecedents of being moved are very similar for many other cultures and languages. This assumption is supported by Tokaji's (2003) study, which explored the emotional state called *kandoh*, a Japanese emotion term that is translated as *being emotionally moved*. In contrast to our study, Tokaji (2003) did not use an approach based on linguistic terms but instead directly asked questions regarding the emotional state. Still, he too found a predominant association of *kandoh* with both sadness and joy and presented evidence that sad events the participants characterized as moving were nevertheless rated as pleasant to some degree.

In summary, our free-association data collected from a large and fairly representative group of participants provide evidence that being moved is conceived of (at least by German native speakers) as a specific emotional state often experienced in response to highly significant life events which typically concern close persons. Art experiences, preeminently those involving music and film, were likewise associated as elicitors of touching and moving experiences. Although the present study has taken a language-based exploratory approach and is thus limited through its very design, it yields a language-guided mapping of the conceptual structure of being moved and thereby has also brought us to a position to raise several crucial questions which await further treatment.

### Conflict of interest statement

The authors declare that the research was conducted in the absence of any commercial or financial relationships that could be construed as a potential conflict of interest.
