# Supplementary material for: Being moved: linguistic representation and conceptual structure
Source: Front Psychol. 2014 Nov 3;5:1242. doi: 10.3389/fpsyg.2014.01242 (PMC4217337; doi:10.3389/fpsyg.2014.01242)
Supplement: Supplementary file 1 [file Table1.PDF]

Date 28.06.2014

Supplementary Material

The feeling of Being Moved: Linguistic Representation and Conceptual Structure

Milena Kuehnast[1, 2] *, Valentin Wagner[2, 3], Eugen Wassiliwizky[2, 3], Thomas Jacobsen[2, 4] & Winfried Menninghaus[2, 3]

[1]Centre for General Linguistics, Berlin, Germany
[2]Cluster of Excellence 'Languages of Emotion', Freie Universität Berlin, Germany
[3]Max Planck Institute for Empirical Aesthetics, Frankfurt am Main, Germany
[4]Helmut Schmidt University / University of the Federal Armed Forces Hamburg, Germany

*** Correspondence:** Milena Kuehnast, Centre for General Linguistics (ZAS), Schuetzenstr. 18, D-10117 Berlin, Germany
kuehnast@zas.gwz-berlin.de

1. Supplementary Figures and Tables

1.1. Supplementary Tables

Supplementary Table 1. Cognitive Salience Index of the associations elicited by present and past participles

Prime		Knowledge type	Association		CSI
bewegend	(moving)				
		linguistic / taxonomic	Gefühl	(feeling)	0,067
		introspective	Freude	(joy)	0,075
			Trauer	(mourning/sadness)	0,070

		Liebe	(love)	0,064
		Tränen	(tears)	0,061
		traurig	(sad)	0,060
		Glück	(happiness)	0,044
		Angst	(fear)	0,025
	situational	Katastrophe	(catastrophe)	0,100
		Familie	(family)	0,065
		Tod	(death)	0,039
		Kinder	(children)	0,038
		Hochzeit	(wedding)	0,033
		Film	(film)	0,032
		Musik	(music)	0,029
		Beziehung	(relationship)	0,027
		Fußball	(football)	0,027
		Geburt	(birth)	0,021
bewegt (moved)				
	linguistic / taxonomic	Gefühl	(feeling)	0,036
	introspective	Liebe	(love)	0,093

	Freude	(joy)	0,086
	Trauer	(mourning/sadness)	0,073
	traurig	(sad)	0,072
	Wut	(rage)	0,052
	Hass	(hate)	0,031
	fröhlich	(cheerful)	0,028
	verliebt	(in love)	0,028
	lachen	(laughing)	0,024
	Spaß	(fun)	0,022
	Glück	(happiness)	0,015
	Tränen	(tears)	0,013
situational	Tod	(death)	0,041
	Film	(film)	0,028
	Geburt	(birth)	0,028
	Unglück	(calamity)	0,026
	Familie	(family)	0,024
	Hochzeit	(wedding)	0,020
	Kinder	(children)	0,018
	Unfall	(accident)	0,018
	Musik	(music)	0,015

berührend (touching)				
	linguistic / taxonomic	Gefühl	(feeling)	0,114
		ergreifend	(deeply moving)	0,059
	introspective	Liebe	(love)	0,160
		traurig	(sad)	0,110
		Freude	(joy)	0,093
		Trauer	(mourning/sadness)	0,091
		Glück	(happiness)	0,046
		Tränen	(tears)	0,043
		Leid	(suffering)	0,035
		weinen	(crying)	0,034
		lachen	(laughing)	0,032
		Empathie	(empathy)	0,029
		lächeln	(smiling)	0,022
		fröhlich	(cheerful)	0,020
	situational	Film	(film)	0,137
		Musik	(music)	0,059
		Kinder	(children)	0,043

			Tod	(death)	0,039
			Katastrophe	(catastrophe)	0,034
			Geburt	(birth)	0,028
			Beziehung	(relationship)	0,025
			Familie	(family)	0,018
			Bilder	(pictures)	0,012
			Schönheit	(beauty)	0,007
berührt	touched				
		linguistic / taxonomic	Gefühl	(feeling)	0,250
			ergriffen	(deeply moved)	0,063
		introspective	traurig	(sad)	0,158
			Liebe	(love)	0,143
			Trauer	(mourning/sadness)	0,080
			Freude	(joy)	0,074
			glücklich	(happy)	0,036
			Leben	(life)	0,031
			Glück	(happiness)	0,019
			weinen	(crying)	0,018
			lachen	(laughing)	0,015

		situational	Kinder	(children)	0,038
			Tod	(death)	0,026
			Schicksal	(fate)	0,023
			Natur	(nature)	0,021
			Familie	(family)	0,020
			Geburt	(birth)	0,017
rührend	(stirring)				
		linguistic	bewegend	(moving)	0,123
			Gefühl	(feeling)	0,077
			ergreifend	(deeply moving)	0,055
		introspective	traurig	(sad)	0,103
			Tränen	(tears)	0,092
			Trauer	(mourning/sadness)	0,087
			Freude	(joy)	0,040
			Glück	(happiness)	0,035
			mitfühlend	(sympathetic)	0,032
			Liebe	(love)	0,031
			weinen	(crying)	0,030
			Mitleid	(compassion)	0,022

		situational	Kinder	(children)	0,103
			Film	(film)	0,052
			Tod	(death)	0,047
			Hochzeit	(wedding)	0,037
			Musik	(music)	0,030
			Wiedersehen	(reunion)	0,028
			Abschied	(farewell)	0,021
			Bücher	(books)	0,021
			Geburt	(birth)	0,017
			Freunde	(friends)	0,014
gerührt	(stirred)				
		linguistic / taxonomic	Tränen	(tears)	0,140
			Gefühl	(feeling)	0,083
			berührt	(touched)	0,052
			ergriffen	(deeply moved)	0,052
			Emotion	(emotion)	0,026
		introspective	Trauer	(mourning/sadness)	0,153
			weinen	(crying)	0,143

			Freude	(joy)	0,116
			lachen	(laughing)	0,103
			Liebe	(love)	0,081
			traurig	(sad)	0,069
			Glück	(happiness)	0,044
			sensibel	(sensitive)	0,026
		situational	Geburt	(birth)	0,041
			Tod	(death)	0,038
			Freunde	(friends)	0,037
			Baby	(baby)	0,035
			Kinder	(children)	0,021
			Menschen	(humans)	0,017
ergreifend	(deeply moving)				
		linguistic / taxonomic	Gefühl	(feeling)	0,130
		introspective	Liebe	(love)	0,128
			traurig	(sad)	0,095
			weinen	(crying)	0,089
			Freude	(joy)	0,067

		Trauer	(mourning(sadness)	0,056
		Tränen	(tears)	0,029
	situational	Geburt	(birth)	0,124
		Familie	(family)	0,100
		Film	(film)	0,093
		Tod	(death)	0,061
		Hochzeit	(wedding)	0,053
		Musik	(music)	0,026
		Kino	cinema	0,020
		Bücher	books	0,011
ergriffen (deeply moved)				
	linguistic / taxonomic	Gefühl	(feeling)	0,083
		bewegt	(moved)	0,025
	introspective	Trauer	(mourning/sadness)	0,142
		Freude	(joy)	0,132
		weinen	(crying)	0,117
		Schmerz	(pain)	0,107
		traurig	(sad)	0,089

		Tränen	(tears)	0,071
		Wut	rage	0,057
		lachen	(laughing)	0,045
		Liebe	(love)	0,032
		Freundschaft	(friendship)	0,010
	situational	Kinder	(children)	0,027
erschütternd (shattering)				
	introspective	traurig	(sad)	0,095
		Trauer	(mourning/sadness)	0,059
		Leid	(suffering)	0,018
		Liebe	(love)	0,016
		Schmerz	(pain)	0,016
		Angst	(fear)	0,010
	situational	Tod	(death)	0,142
		Fukushima	(Fukushima)	0,097
		Erdbeben	(earthquake)	0,086
		Trennung	(parting)	0,077
		Katastrophe	(catastrophe)	0,035
		Krankheit	(sickness)	0,032

		Verlust	(loss)	0,032
		Krieg	(war)	0,029
		Unfall	(accident)	0,019
erschüttert (shattered)				
	linguistic / taxonomic	Gefühl	(feeling)	0,058
	introspective	erschrocken	(terrified)	0,035
		traurig	(sad)	0,248
		Trauer	(mourning/sadness)	0,190
		enttäuscht	(disappointed)	0,069
		verunsichert	(insecure)	0,052
		Traurigkeit	(sadness)	0,041
		Schmerz	(pain)	0,035
		Tränen	(tears)	0,035
		verletzt	(hurt)	0,030
		Freude	(joy)	0,023
		verzweifelt	(desperate)	0,021
		Angst	(fear)	0,015
	situational	Erdbeben	(earthquake)	0,039

			Familie	(family)	0,035
			Katastrophe	(catastrophe)	0,035
			Tod	(death)	0,031
			Japan	(Japan)	0,030
			Verlust	(loss)	0,029
			Veränderung	(change)	0,017
erhebend	(elevating)				
		linguistic / taxonomic	Gefühl	(feeling)	0,148
		introspective	Freude	(joy)	0,155
			Glück	(happiness)	0,089
			Wut	(rage)	0,069
			Liebe	(love)	0,062
			Freundschaft	(friendship)	0,052
		situational	Sonne	(sun)	0,073
			Urlaub	(holiday)	0,032
			Erfolg	(success)	0,023
			Familie	(family)	0,022
			Meer	(sea)	0,021

			Musik	(music)	0,014
erhoben	(elevated)				
		linguistic / taxonomic	Gefühl	(feeling)	0,092
		introspective	Freude	(joy)	0,063
			Trauer	(mourning/sadness)	0,042
			Wut	(rage)	0,039
			Glück	(happiness)	0,038
			Liebe	(love)	0,038
			glücklich	(happy)	0,035
		situational	Sieg	(victory)	0,026
			Kinder	(children)	0,016
packend	(gripping)				
		linguistic / taxonomic	ergreifend	(deeply moving)	0,043
			Gefühl	(feeling)	0,027
		introspective	Liebe	(love)	0,080
			traurig	(sad)	0,044
			Freude	(joy)	0,041

		weinen	(crying)	0,035
		Hass	(hate)	0,025
		lachen	(laughing)	0,025
	situational	Film	(film)	0,078
		Theater	(theatre)	0,030
		Streit	(argument)	0,019
gepackt	(gripped)			
	linguistic / taxonomic	Gefühl	(feeling)	0,310
		ergriffen	(deeply moved)	0,129
		gefesselt	(riveted)	0,065
		berührt	(touched)	0,039
	introspective	begeistert	(enthusiastic)	0,032
		erregt	(thrilled)	0,022
		Freude	(joy)	0,048
		verliebt	(in love)	0,032
		wütend	(furious)	0,032
		Liebe	(love)	0,030
		Spannung	(suspense)	0,028

			Trauer	(mourning/sadness)	0,028
			lachen	(laughing)	0,021
			Angst	(fear)	0,018
			weinen	(crying)	0,017
		situational	Film	(film)	0,043
aufregend	(exciting)				
		linguistic / taxonomic	Gefühl	(feeling)	0,042
		introspective	Wut	(rage)	0,065
			Liebe	(love)	0,061
			Spannung	(suspense)	0,055
			Trauer	(mourning/sadness)	0,054
			Freude	(joy)	0,041
			Hass	(hate)	0,030
			Ärger	(anger)	0,029
			Angst	(fear)	0,024
			Schmerz	(pain)	0,024
			Stress	(stress)	0,024
		situational	Streit	(argument)	0,054

		Sex	(sex)	0,048
		Gefahr	(danger)	0,029
		Geburt	(birth)	0,022
		Hochzeit	(wedding)	0,022
		Musik	(music)	0,022
		Tod	(death)	0,016
aufgeregt (excited)				
	linguistic / taxonomic	nervös	(nervous)	0,097
	introspective	unruhig	(anxious)	0,065
		Liebe	(love)	0,060
		Freude	(joy)	0,054
		wütend	(furious)	0,052
		Wut	(rage)	0,048
		aufgewühlt	(agitated)	0,047
		angespannt	(tense)	0,043
		Angst	(fear)	0,034
		zittern	(shiver)	0,024
		unsicher	(uncertain)	0,022

	Spannung	(suspense)	0,018
	weinen	(crying)	0,016
	Anspannung	(tension)	0,015
	Ärger	(anger)	0,011
	Vorfreude	(anticipation)	0,011
situational	Sex	(sex)	0,032
	Prüfung	(exam)	0,024
	Familie	(family)	0,011

Notes. The associations are organised according to the three knowledge types adopted from Wu & Barsalou's (2009) taxonomy: linguistic & taxonomic relations, situational features and introspective features. English translations are given in brackets.

Supplementary Table 2. Overlapping Coefficient matrix

German primes	English translation	Item Nr.	1	2	3	4	5	6	7	8	9	10	11	12	13	14	15	16
Past participles																		
bewegt	(moved)	1																
berührt	(touched)	2	.567															
gerührt	(stirred)	3	.519	.608														
ergriffen	(deeply moved)	4	.443	.566	.553													
erschüttert	(shattered)	5	.308	.366	.322	.398												
erhoben	(elevated)	6	.416	.512	.398	.509	.202											
gepackt	(gripped)	7	.393	.458	.415	.388	.181	.336										
aufgeregt	(excited)	8	.258	.251	.204	.218	.107	.221	.311									
Present participles																		
bewegend	(moving)	9	**.628**	.513	.517	.383	.407	.419	.363	.235								
berührend	(touching)	10	.661	**.609**	.566	.456	.319	.407	.433	.223	.679							
rührend	(stirring)	11	.528	.477	**.553**	.404	.321	.301	.302	.136	.597	.570						
ergreifend	(deeply moving)	12	.633	.524	.492	**.386**	.343	.295	.380	.223	.604	.649	.632					
erschütternd	(shattering)	13	.254	.289	.197	.268	**.432**	.119	.143	.090	.281	.240	.226	.240				
erhebend	(elevating)	14	.400	.369	.262	.379	.148	**.523**	.259	.260	.399	.384	.214	.325	.045			
packend	(gripping)	15	.433	.483	.420	.483	.173	.311	**.399**	.221	.323	.464	.350	.452	.126	.323		
aufregend	(exciting)	16	.534	.383	.349	.322	.253	.335	.327	**.378**	.466	.424	.358	.432	.247	.316	.351	

Note. The values printed in bold represent the overlapping coefficient between the participle forms of each verb.

1.2. Supplementary Figures

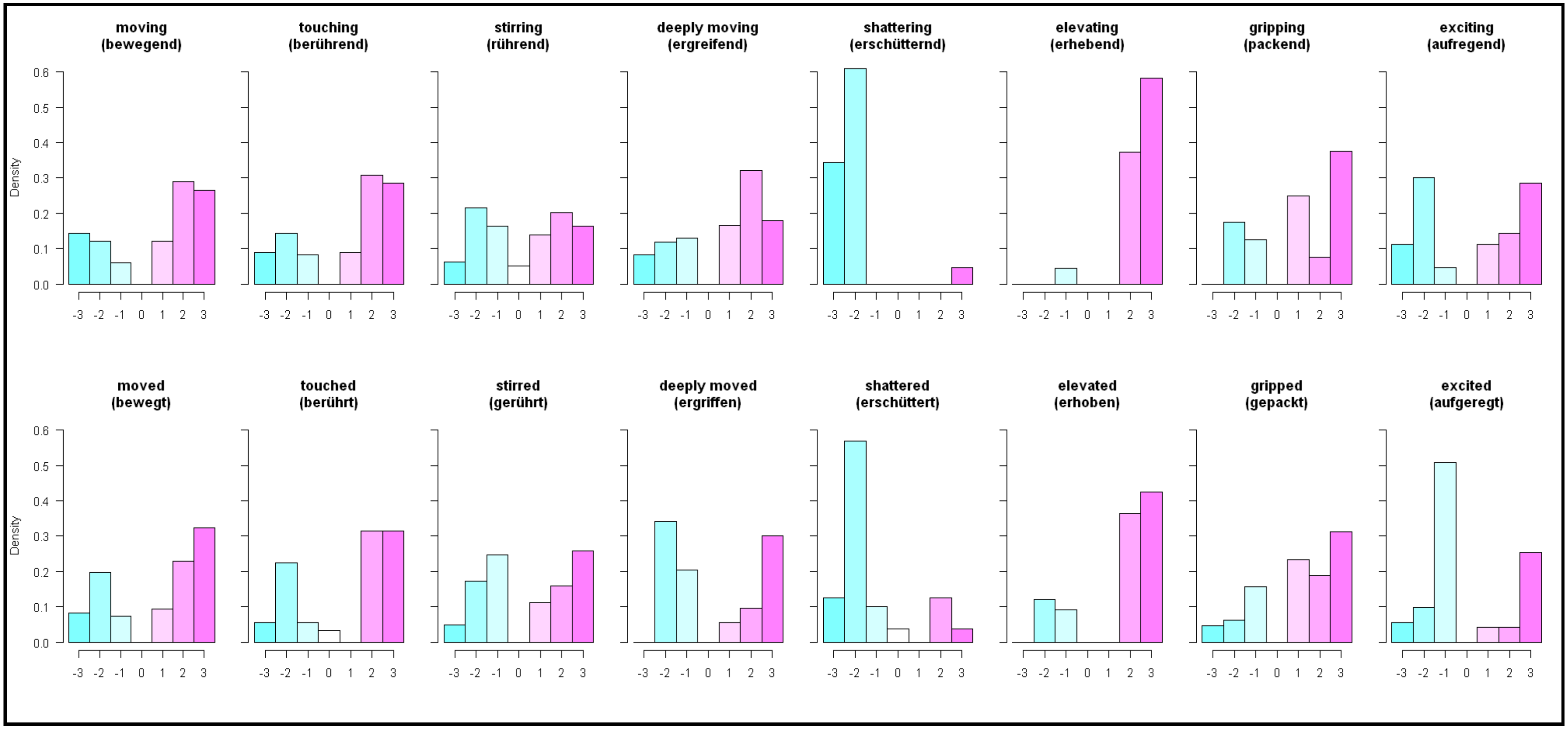

Supplementary Figure 1. Bimodal distribution of positive and negative associations obtained for each participle.

2. References

Wu, L.-l., & Barsalou, L. W. (2009). Perceptual simulation in conceptual combination: Evidence from property generation. *Acta Psychologica*, *132*, 173–189. doi: 10.1016/j.actpsy.2009.02.002
